# A situational and stakeholder analysis of health technology assessment in Zimbabwe

**DOI:** 10.1017/S0266462324000266

**Published:** 2024-04-29

**Authors:** Blessing Dzingirai, Prudence Dambiko, Celia Matyanga, Pinky Manyau, Dexter Tagwireyi, Maarten J. Postma, Nyashadzaishe Mafirakureva, Marinus van Hulst

**Affiliations:** 1 Department of Pharmacy and Pharmaceutical Sciences, University of Zimbabwe, Harare, Zimbabwe; 2 Department of Economics, Econometrics & Finance, Faculty of Economics and Business, University of Groningen, Groningen, The Netherlands; 3 Department of Health Sciences, University of Groningen, University Medical Center Groningen, Groningen, The Netherlands; 4 Health Economics and Decision Science, School of Health and Related Research, University of Sheffield, UK; 5 Department of Clinical Pharmacy and Toxicology, Martini Hospital, Groningen, The Netherlands

**Keywords:** health technology assessment, priority setting, stakeholder participation, low and middle income countries, Universal Health Coverage, Zimbabwe

## Abstract

**Objectives:**

Systematic priority setting is necessary for achieving high-quality healthcare using limited resources in low- and middle-income countries. Health technology assessment (HTA) is a tool that can be used for systematic priority setting. The objective of this study was to conduct a stakeholder and situational analysis of HTA in Zimbabwe.

**Methods:**

We identified and analyzed stakeholders using the International Decision Support Initiative checklist. The identified stakeholders were invited to an HTA workshop convened at the University of Zimbabwe. We used an existing HTA situational analysis questionnaire to ask for participants’ views on the need, demand, and supply of HTA. A follow-up survey was done among representatives of stakeholder organizations that failed to attend the workshop. We reviewed two health policy documents relevant to the HTA. Qualitative data from the survey and document review were analyzed using thematic analysis.

**Results:**

Forty-eight organizations were identified as stakeholders for HTA in Zimbabwe. A total of 41 respondents from these stakeholder organizations participated in the survey. Respondents highlighted that the HTA was needed for transparent decision making. The demand for HTA-related evidence was high except for the health economic and ethics dimensions, perhaps reflecting a lack of awareness. Ministry of Health was listed as a major supplier of HTA data.

**Conclusions:**

There is no formal HTA agency in the Zimbabwe healthcare system. Various institutions make decisions on prioritization, procurement, and coverage of health services. The activities undertaken by these organizations provide context for the institutionalization of HTA in Zimbabwe.

## Introduction

The Zimbabwean government adopted the universal health coverage (UHC) political declaration in 2019 and aims to achieve UHC by 2030 (1). The critical concepts of UHC require information on the range of health services to be provided, the population to be covered, and financial protection (2). Zimbabwe must define its own UHC pathway by generating these key data based on the health needs of the population and available resources. Health technology assessment (HTA) is increasingly being used to inform decisions in the UHC context (3;4). HTA has been used to aid in priority setting, formulate essential medicine lists, establish treatment guidelines, establish essential health packages, and identify health interventions that provide the best value in similar economic settings as Zimbabwe (5–7). HTA can be a very important tool for achieving UHC goals in Zimbabwe.

HTA is a multidisciplinary process in which explicit methods are used to determine the value of health technology at different points in its lifecycle (8). In 2014, the World Health Organization (WHO) encouraged member states to establish national HTA systems to support policy decisions (9). Despite the recommendation from the WHO and the great need, very few low- and middle-income countries (LMICs) have institutionalized HTA (10;11). The major challenges associated with the institutionalization of HTA in LMICs, include lack of expertise and awareness, a paucity of local utility and unit cost data, and a lack of political will (10;11). Despite these challenges, some LMICs (Ethiopia, Ghana, Tanzania, and Kenya) have initiated HTA activities with donor support (6;7;12;13). For example, Tanzania has created an HTA committee that has revised its essential medicine list and treatment guidelines (12). Zimbabwe can draw lessons from countries that have begun the HTA journey.

The Zimbabwean healthcare system is composed of public institutions supported by private health facilities, local authority clinics, and church-based health institutions (14). The public healthcare system has five tiers and operates on a referral basis from the lowest to the highest level. The levels of care are primary (rural health facilities and private general practitioners), secondary (district hospitals), tertiary (provincial hospitals), quaternary (specialist services and medical schools teaching hospitals), and quinary (research and development hospitals linked to universities). The government, external funders, private insurance, and out-of-pocket expenditures fund the healthcare sector (14). The government funding, currently at 11 percent of the total national budget in 2023, falls short of the Abuja Declaration target (15). As a result of low government funding, there is dependence on external funding, which averaged 60 percent of the total health expenditure for 2014–2021 (16). In addition to limited government expenditures on health, Zimbabwe does not operate a mandatory socialized health insurance system, and private health insurance is very low covering only around 10 percent (14).

In a guidance document for setting up HTA in LMICs, the Management Sciences for Health recommended a model that involves agenda setting, policy formulation, adoption/implementation, and impact evaluation (17). Various models may be used in the agenda-setting process for the introduction of HTA, such as the stakeholder analysis model (17) and Kingdon’s model of policy analysis (18), content, context, and process (17). For example, Kingdon’s model states that a window of opportunity for HTA introduction occurs when the problem, policy, and politics around priority setting in healthcare converge (17). All three models highlight context consideration as pivotal to successfully implementing HTA. The contextual aspects that need to be defined for the institutionalization of HTA include the fiscal environment, health systems, regulation, and stakeholders. Situational and stakeholder analyses are vital inputs in the agenda-setting step of HTA introduction. The objective of this study was to conduct situational and stakeholder analyses to inform the future institutionalization of HTA in Zimbabwe.

## Methods

### Stakeholder mapping

Stakeholder mapping and analysis were independently performed by two researchers using a checklist developed by the International Decision Supportive Initiative (iDSI) (19). This checklist has recently been used to determine relevant HTA stakeholders in the Egyptian context (20), and it characterizes stakeholders into nodes, networks and, environments, based on the capacity-building framework of the iDSI (21). For each category of stakeholders, the tool suggests a set of questions that help identify relevant stakeholders for a particular country. The results of the mapping exercise were compared between the researchers and discussion was used to reach consensus in case of differences.

### Data collection

We convened an HTA workshop at the University of Zimbabwe in July 2019. The stakeholders identified from the mapping exercise were invited to attend the workshop. Presentations focusing on HTA were given at the workshop by a health economist from the University of Sheffield (UK). Other presentations were given on how decisions are made to produce essential medicine lists and treatment guidelines, establish health priorities, and determine health tariffs in Zimbabwe. After the workshop, we surveyed the workshop participants using the adapted questionnaire developed by Health Interventions and Technology Assessment (HITAP) and the National Institute of Health and Care Excellence (NICE) International (20). An adapted version of the questionnaire was used in similar studies in Uganda and Nigeria (22;23). The conceptual framework of the questionnaire describes three elements (i) the need for HTA, (ii) the demand for HTA, and (iii) the supply for HTA. We also distributed the questionnaire to stakeholders who were not represented at the workshop as a follow-up survey. We also reviewed the National Health Strategy (NHS) 2021–2025 and the Zimbabwe Health Financing Strategy (HFS) 2017 documents (1;14). The selection of the documents was informed by previous research that explored health policy documents in Zimbabwe (24).

### Data analysis

Qualitative data from the survey and strategic document review were analyzed using thematic analysis (25). We utilized the predefined themes as informed by the HITAP-NICE HTA conceptual framework to carry out deductive, structural coding (23). The themes were as follows: current HTA activities in Zimbabwe, the need for HTA, the demand for HTA, the supply of HTA, and the challenges in institutionalizing HTA in Zimbabwe. Two of the researchers read through the transcripts and coded the data into predefined themes by answering the questions “who,” “what,” “where,” and “how.” The thematic coding tree for the qualitative data is shown in Supplementary Figure S1. All data recorded under the themes were used for the write-up.

### Ethical approval

Ethical approval to conduct the study was granted by the Joint Research Ethics Committee for University of Zimbabwe College of Health Sciences and Parirenyatwa Hospitals (JREC/89/19). The study participants signed informed consent forms before taking part in the survey.

## Results

### Stakeholder mapping

A total of 48 stakeholders (organizations) were identified as key to the HTA process in Zimbabwe. A summary of all the stakeholders identified to be relevant to the introduction of HTA in Zimbabwe is shown in Supplementary Table S1. A total of 33 participants attended the workshop and participated in the survey. An additional 8 key informants participated in the follow-up survey for a total of 41 respondents for this study. The organizations represented by the participants are shown in Table 1.

**Table 1.** Summary of the study participants

Organization	Number	Percent (*N* = 41)	Attended the workshop
Ministry of Health and Child Care	3	7.3	Yes
University of Zimbabwe	10	24.5	Yes
Medicines Control Authority of Zimbabwe	2	4.9	No
National Medicine and Therapeutics Policy Advisory Committee	1	2.4	Yes
Department of Pharmacy Services, Ministry of Health and Child Care	3	7.3	Yes
National Blood Services of Zimbabwe	3	7.3	Yes
Retail Pharmacies Association of Zimbabwe	5	12.3	Yes
Association of Health Funders of Zimbabwe	3	7.3	Yes
CIMAS Medical Aid Society	3	7.3	Yes
Premier Medical Aid Service	3	7.3	Yes
Varichem (Private Pharmaceutical Manufacturer)	1	2.4	No
National Social Security Authority	1	2.4	No
Research Institutions	3	7.3	Yes

### Survey results

#### Current HTA activities in Zimbabwe

At the time of the study, there was no formal institution that performed coordinated and explicit HTA processes in Zimbabwe. However, from the presentations at the workshop and the document review, we obtained information on how various organizations perform activities that aid decision making on priority setting, market authorization of medicines, developing essential medicine lists and treatment guidelines, and reimbursement. These activities provide context for HTA institutionalization.

#### Ministry of Health and Child Care

The Ministry of Health and Child Care (MoHCC) provides coordination and regulatory roles to all health institutions in Zimbabwe. The MoHCC sets health sector priorities for every 5-year cycle using NHS documents. The MoHCC, through the 2021–2025 NHS aims to “*provide, administer, coordinate, promote and advocate for the provision of equitable, appropriate, accessible, affordable, and acceptable quality health services and care to Zimbabweans while maximizing the use of available resources”* (1). The MoHCC priority setting process is informed through consultation with stakeholders, information from routine surveillance, and surveys by the MoHCC and Sustainable Development Goals. Stakeholders involved in priority setting include academic institutions, other government ministries with roles in health (e.g., the Ministry of Finance), health profession councils, private health providers, health insurance providers, traditional leaders, and development partners. Within the NHS, the MoHCC defined the essential health services package. Currently, essential health service packages are defined for primary and secondary tiers of public healthcare and the MoHCC plans to define packages for the tertiary and quaternary tiers. The MoHCC is also responsible for allocating financial resources to healthcare interventions, another key activity where HTA can be used. The MoHCC utilizes program-based budgeting (PBB), results-based financing (RBF), and need-based resource allocation frameworks to allocate financial resources in the public healthcare system. The PBB was introduced in 2017 and links spending to health outcomes. Under the PBB, the MoHCC defined four programs namely policy and administration, public health, primary and hospital care and biomedical engineering, and pharmaceuticals. The objectives and expected outcomes of every program are defined and funding is allocated to the programs with the greatest health impact. The RBF was introduced in 2011 and involved reimbursing district hospitals after achieving preset outcomes in maternal and child health services. Needs-based resource allocation involves allocating funds based on geographical health indicators such as population size. The review of the HFS also revealed challenges in resource allocation that included a lack of transparency, accountability, and weak procurement systems. The Zimbabwean government intends to establish a National Health Insurance (NHI) system, as outlined in the NHS 2021–2025. The government’s rationale for establishing the NHI is to ensure equitable health financing and protect people from out-of-pocket payments for health. At the time of this study, no NHI had been established in the Zimbabwean health system.

#### Essential medicines list and treatment guidelines

The MoHCC established the National Medicine and Therapeutics Policy Advisory Committee (NMTPAC), which is responsible for the development and periodic review of the Essential Medicines List and Standard Treatment Guidelines of Zimbabwe (EDLIZ). The committee consists of medical doctors and pharmacists working voluntarily. In addition to the selection of medicines for inclusion, the EDLIZ is also used to classify medicines in terms of priority for availability. For example, some medicines are categorized as vital (V) and are supposed to be available at all public health institutions. The EDLIZ is also a tool used to determine coverage of access to medicines and health services. For example, some medicines are coded B medicines and can be accessed only at the district hospital level (secondary care tier) and above. The classification of medicines by level of availability is based on the availability of expertise and diagnostic tests to support the administration of the medicines at different levels of care. The NMTPAC considers evidence on relevance to disease burden, efficacy, quality, cost, and potential for local manufacture as criteria for the inclusion of medicines in the EDLIZ. Although cost-effectiveness is listed in the EDLIZ as one of the criteria for drug inclusion, cost-effectiveness analysis evidence is currently not used to inform the selection of medicines.

#### Market authorization of medicines

The Medicines Control Authority of Zimbabwe (MCAZ) is mandated by an act of parliament to register and provide market authorization for medicines before they can be accessed for use in Zimbabwe. The MCAZ considers evidence on efficacy, safety, and quality submitted as a dossier by the applicants (manufacturers or their representatives). The MCAZ also makes decisions on the removal of medicines from the register based on a lack of effectiveness or safety issues. To accomplish this, the MCAZ collects data on adverse events from the general public and health professionals using post-marketing surveillance frameworks.

### Private players

The private players in the healthcare sector in Zimbabwe include private health providers and medical insurance institutions (medical aid societies). Medical health insurance companies are registered with the Association of Healthcare Funders of Zimbabwe (AFHOZ). Private health providers must also register with the AFHOZ for their claims to be reimbursed by medical insurance companies. The AFHOZ sets tariffs for health services. AFHOZ’s presentation at the workshop revealed that they were in the process of implementing a new framework for determining tariffs as a way to resolve tariff inequalities. The new tariff schedule is based on the resource-based relative value scale (RBRVS). The RBRVS tariff is a product of a relative value unit (RVU) and a conversion factor for each health service. The RVU accounts for health professional expertise, the time used to provide the service, and the cost of maintaining the practice. Stakeholders consulted in developing the new tariff system included health professionals and medical insurance companies. Private healthcare providers are also collecting valuable data for HTA, such as drug utilization and coverage of health interventions. One of the challenges highlighted by the respondents is situations in which healthcare funders/purchasers assume provider roles. For example, some private health insurance players are involved in providing clinical and pharmaceutical services, potentially, resulting in a distorted valuation of health interventions because of potential conflicting interests. Furthermore, they highlighted discrepancies between private healthcare providers’ tariffs and what healthcare funders agree to reimburse resulting in patients having to pay the resulting shortfalls. These out-of-pocket payments of shortfalls may expose individuals to potential catastrophic health expenditures.

### Academic and research institutions

The Medical Research Council of Zimbabwe oversees all health research and ethics in health research in Zimbabwe. Various academic and research institutions performed research and generated evidence on disease burden, coverage and effectiveness of health interventions, health-related quality of life, costs, and cost-effectiveness. For example, researchers from the National Blood Service of Zimbabwe presented a paper on the cost-effectiveness of adding nucleic acid testing in screening blood in Zimbabwe during the workshop (26). This finding clearly showed that HTA can aid in blood safety decisions in Zimbabwe.

### The need for HTA

**Figure 1.**
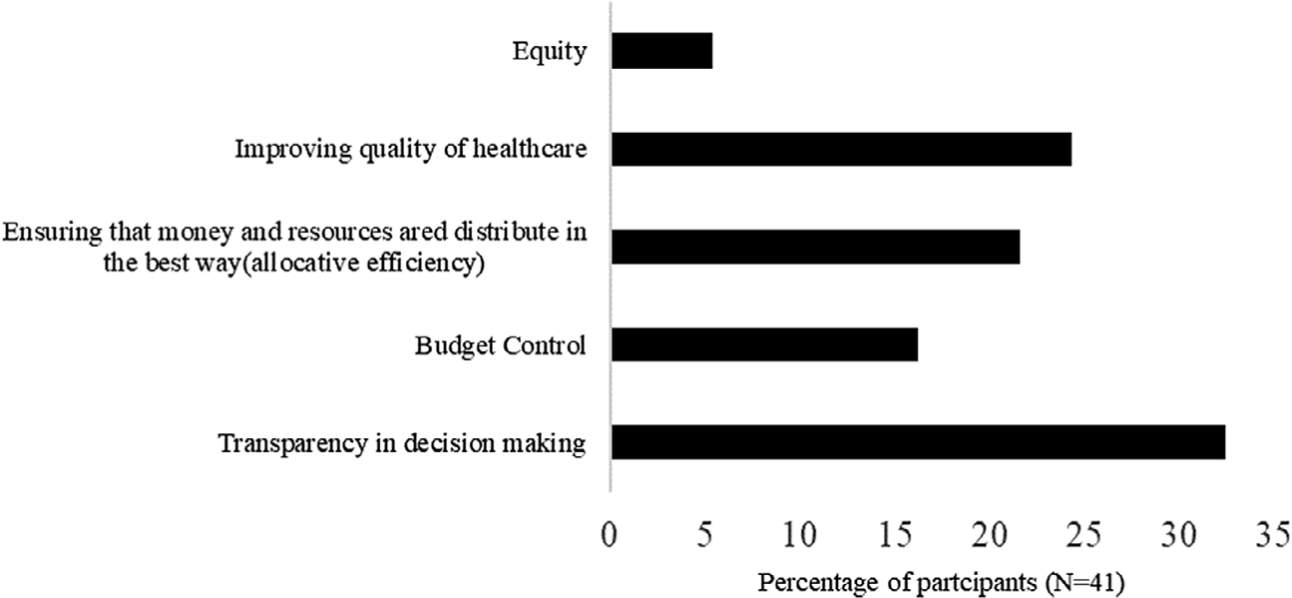
Attributes of health technology assessment that were perceived as important for Zimbabwe.

**Figure 2.**
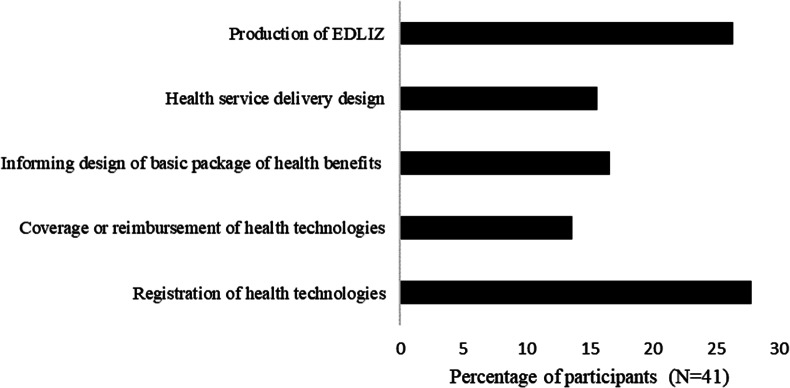
The policy areas where health technology assessment is needed in Zimbabwe. EDLIZ, Essential Medicines List and Standard Treatment Guidelines for Zimbabwe.

Respondents to the study listed the attributes of HTA that were important to the Zimbabwean context and policy areas that needed HTA in Zimbabwe. The responses are presented in Figures 1 and 2. Most respondents to the survey reported that the capability of HTA to increase transparency (32 percent) followed by to improve the quality of health (24 percent) were the most important attributes. Most respondents suggested that HTA was needed more for the registration of health technologies (27 percent) and for the production of the essential medicine lists and treatment guidelines (26 percent).

### The demand for HTA

**Figure 3.**
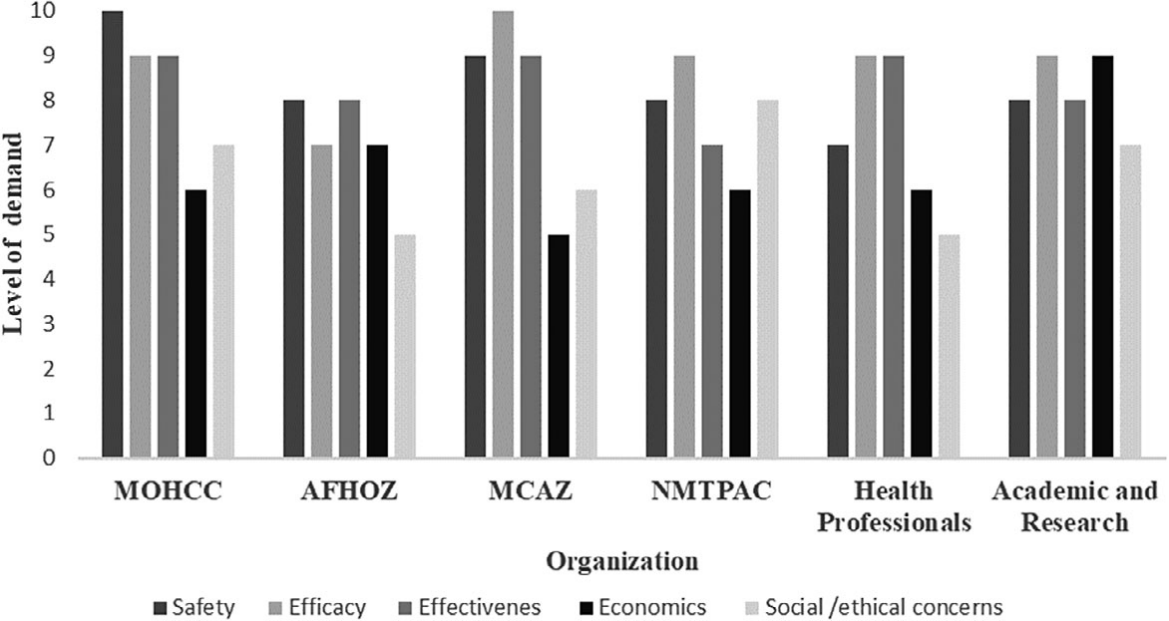
The potential users of health technology assessment output and the perceived levels of demand for evidence. AFHOZ, Association of Healthcare Funders of Zimbabwe; MCAZ, Medicines Control Authority of Zimbabwe; MOHCC, Ministry of Health and Child Care; NMTPAC, National Medicine and Therapeutics Policy Advisory Committee.

The respondents were asked to identify potential users of HTA output in Zimbabwe and indicate their perceived level of demand on a scale of 0–10, where 0 represented no demand and 10 indicated high demand. The organizations that were identified as potential users of HTA outputs and the average scores for the perceived level of demand are presented in Figure 3. The level of demand for all types of evidence was high except for economics and social/ethical evidence, which had scores below 5.

### The supply of HTA

The Zimbabwe Demographic Health Survey was identified a source of demographic information as well as health services utilization and health indicators data. The MoHCC health information system was listed as a source of data on disease burden, unit costs, and health outcomes. Research institutes were listed as sources of clinical effectiveness data. Supplementary Table S2 summarizes the potential data sources for HTA in Zimbabwe.

### Challenges to the implementation of HTA

Several potential challenges to introducing institutionalized HTA in Zimbabwe were identified from the survey. The major challenge highlighted by the participants was the lack of financial resources. A greater part of the government expenditure on health is spent on salaries leaving very little for patient care. The lack of local health economic evaluation expertise to successfully implement HTA was highlighted as another barrier. The number of health economists in the country is very small mainly because there are no universities that offer health economics training. Most of the participants were willing to send their staff for training in skills relevant to HTA processes and methods. We also noted a paucity of data on unit costs, health-related quality of life scores, and outcomes of health interventions.

## Discussion

To the best of the author’s knowledge, this was the first study to explore the situational analysis of HTA in Zimbabwe. Despite the absence of an HTA agency, there are formal decision-making processes characterized by the consideration of scientific evidence and multidisciplinary consultations in the Zimbabwe healthcare system. Examples include the processes involved in developing the NHS by the MoHCC, the essential medicines list by the NMTPAC, and the registration of medicines by the MCAZ. A multidisciplinary decision-making approach is a crucial aspect of HTA and provides a strong platform conducive to introducing HTA. The challenges in the Zimbabwean health system (lack of health personnel, medicines, and funding (27–29)), which represent the problem stream of Kingdon’s model, can be leveraged to advocate for the implementation of HTA.

Stakeholders relevant to the HTA processes in Zimbabwe were identified in this study. Further analysis of the stakeholders is required to establish their position, power, and views regarding HTA. This approach is important for determining the level of engagement required to build consensus and political will for HTA (30). The key stakeholders that drive political will for HTA introduction in Zimbabwe’s healthcare system are the Parliament and MOHCC because they are responsible for enacting and implementing the legislation, respectively (24). Additionally, it is important to involve academic institutions and professionals in the formative stages of HTA institutionalization (31). HTA processes based or affiliated with academic institutions have the advantages of established scientific rigor and a positive perception of authenticity by the public (32). Examples of academic institution engagement in HTA include HTA agencies based at academic institutions, contracted academic institutions, and technical working groups. Zimbabwe has several universities that can engage in various ways to drive HTA. However, there is a need to identify institutions that have the capacity for HTA processes. Other key stakeholders in the introduction of HTA in Zimbabwe are developmental partners such as WHO, UN, and UNICEF. Developmental partners are important because they contribute a substantial proportion of the healthcare funding in Zimbabwe (16) and are potential sources of funding for capacity building.

The need for public and patient involvement in priority setting in healthcare is an important element of HTA and should be carefully considered in Zimbabwe. Public and patient involvement is important for capturing experiences of living with a disease or condition, and the impact of a technology that would otherwise not be obtained from the available literature and expert knowledge (33;34). Patient and public involvement should go beyond mere representation on decision-making committees by equipping individuals to understand and analyze technical evidence on health interventions. Lessons can be drawn from Brazil, where the HTA agency (CONITEC) produced a lay technical report for trastuzumab for public consultation before registration (35). Some HTA agencies have moved further and developed tools to capture and include patient and public views in frameworks to determine the value of health technologies (36;37). All these examples are useful for informing public and patient involvement initiatives in the Zimbabwean context.

The need for HTA in Zimbabwe was highlighted by a plan to roll out an NHI, existing conflicts of interest in the valuation of health services, out-of-pocket expenditures to cover shortfalls, and policy areas that require HTA evidence. HTA is needed to support the efficient implementation of the NHI. HTA has a potential role in defining the health packages to be covered and the levels of reimbursement that are acceptable. Valuable lessons on how to use HTA to inform prioritization can be drawn from South Africa (38) and Ethiopia (7). In South Africa, the government has embarked on setting up an institutionalized HTA agency as part of implementing an NHI. In Ethiopia, the Ministry of Health defined the essential health services package by assigning priority scores to health interventions using seven criteria, which included disease burden, cost-effectiveness, budget impact, equity, financial risk protection, public acceptability, and political acceptability (7). A study by Hansen and Chapman provides another approach for priority setting. Hansen and Chapman estimated the costs and benefits of 65 health interventions in Zimbabwe and ranked them based on cost per disability-adjusted life years averted (39).

The respondents suggested that Zimbabwe would benefit from the transparency attribute of HTA. This reflects an important area of weakness in the current healthcare decision making in Zimbabwe. HTA is characterized by explicit and predetermined frameworks used to determine the value of health services and can be useful for enhancing transparency (40;41). In addition to transparency, allocative efficiency and improving the quality of healthcare were also identified as important attributes of HTA. This was consistent with results from similar studies in Nigeria (23) and Uganda (22). A potential explanation is that allocative efficiency and quality are key aspects of UHC (42) and with the country focusing on achieving UHC, participants may be aware of these aspects. Additionally, quality and efficiency were emphasized in the health policy documents that were reviewed in this study; hence, the participants were knowledgeable about their importance in healthcare.

The policy areas with a potential need for HTA and the corresponding organizations that can use HTA outputs in Zimbabwe were identified. The policy areas and organizations that need HTA identified in this study were similar to those reported in studies carried out in Nigeria and Egypt (20;23). However, in this study, lower levels of demand for health economic and social/ethical evidence were reported across all the listed organizations. This can be explained by the low levels of awareness of how health economics and ethics can be incorporated into decision making.

Zimbabwe faces similar challenges as other LMICs in implementing HTA, such as limited resources, expertise, and data (10;11). One way to overcome the lack of financial resources is providing evidence to justify government investment in HTA. An investment case for HTA can be useful for convincing political leaders of health to invest in HTA. In addition to government investment, international partners such as the iDSI can be considered in the provision of financial and technical support for the introduction of HTA (43). For example, iDSI provided financial and technical support for HTA in Ghana (44) and South Africa (45). The lack of data can be overcome by incorporating data collection into the routine management of patients. For example, community pharmacies can provide drug utilization and cost data from their dispensing records. Zimbabwe has quality of life weights for the EuroQol 5 dimension (EQ-5D) (46), which are important for estimating utilities in health economic evaluations. A review of the study that developed the EQ-5D tariff for Zimbabwe showed very low utilization of the data in Zimbabwe, maybe due to low awareness.

## Limitations

The main limitation of this study was that the knowledge of HTA among the participants was not assessed before the survey. Knowledge of HTA may impact one’s response to the survey. The other limitation was that most of the participants were drawn from the capital city where the administration offices of the key institutions are based. The patient groups were also not represented in the study. Despite these limitations, the results of this study are useful for obtaining a picture of HTA in Zimbabwe.

## Conclusions

There is no formal HTA agency in the Zimbabwe healthcare system. The stakeholders who participated in the study indicated that introducing HTA in the Zimbabwean health system is required to increase transparency, quality, and efficiency in decision making. HTA is also currently needed to support the establishment of NHI by the government in order to achieve UHC. Formal HTA can be instituted to help in decision making in the policy areas identified in this study. Stakeholders identified in the study are key in constituting an HTA agency, formulating HTA frameworks, and building local capacity for HTA.

## Supporting information

Dzingirai et al. supplementary material Dzingirai et al. supplementary material

## Data Availability

The data sets used and/or analyzed during the current study are available from the corresponding author upon request.

## Abbreviations

AFHOZ: Association of Health Funders of Zimbabwe
EDLIZ: Essential Medicines List and Standard Treatment Guidelines of Zimbabwe
HFS: Health Finance Strategy
HTA: health technology assessment
HITAP: Health Interventions and Technology assessment
iDSI: International Decision Supportive Initiative
LMICs: low- and middle-income countries
MCAZ: Medicines Control Authority of Zimbabwe
MoHCC: Ministry of Health and Child Care
MRCZ: Medical Research Council of Zimbabwe
NHI: National Health Insurance
NHS: National Health Strategy
NICE: National Institute of Health and Care Excellence
NMTPAC: National Medicine and Therapeutics Policy Advisory Committee
PBB: program-based budgeting
RBF: results-based financing
RBRVS: resource-based relative value scale
RVU: relative value unit
UHC: universal health coverage

## Supplementary material

The supplementary material for this article can be found at http://doi.org/10.1017/S0266462324000266.
