# Supplementary material for: A situational and stakeholder analysis of health technology assessment in Zimbabwe
Source: Int J Technol Assess Health Care. 2024 Apr 29;40(1):e27. doi: 10.1017/S0266462324000266 (PMC11569906; doi:10.1017/S0266462324000266)
Supplement: Dzingirai et al. supplementary material [file S0266462324000266sup001.docx]

**Supplementary file 1**

**Table S1: The key stakeholders who are likely to have a role in health technology assessment in Zimbabwe**

| **Stakeholder Category** | **Type** | **Stakeholder (s)** | **Comment(why relevant to HTA)** |
| --- | --- | --- | --- |
| **Consumers of evidence** | Political and Politically appointed | Parliament of Zimbabwe | The parliament include ruling and opposition members that enact laws that govern health policies |
|  |  | President | President approves the laws enacted by the parliament which includes laws that govern health eg Public Health Act |
|  |  | Ministry of Health and Child Care (MoHCC) | Ministry of Health and child Care formulates and implement health policies |
|  |  | Ministry of Finance | Ministry of Finance collects and administers funding and regulates procurement though the yearly budget. |
|  | Public Institutions | National Institute of Health Research |  |
|  |  | National Medicine and Therapeutics Policy Advisory Committee (NMTPAC) | Committee of experts who determine the essential medicines list and the treatment guidelines |
|  |  | National Pharmaceutical Company of Zimbabwe (NATPHARM) | The central medicines procurement, storage and distribution unit of the MoHCC |
|  |  | National Reference Laboratory (NRL) | Coordinates national laboratory procurement. |
|  |  | Medicines Control Authority of Zimbabwe (MCAZ) | Provide regulatory role in the registration of medicines and clinical trials. |
|  |  | National Blood Service of Zimbabwe (NBSZ) | NBSZ collects, process and distribute blood and blood products. |
|  |  | Zimbabwe National Family Planning Council (ZNFPC) | ZNFPC is responsible for procurement and distribution of family planning products and services. |
|  | Health Services Managers | Parirenyatwa Hospital, Mpilo Hospital, Harare Central Hospital, Chitungwiza Central hospital, Avenues Clinic, Westend hospital | Public and private most prestigious hospital managing many and complex clinical conditions. |
|  | Health Professional Groups | Health Professions Authority (HPA)  Pharmacist Council of Zimbabwe (PCZ)  Medical and Dental Practitioners of Zimbabwe (MDPZ)  Nurses Council of Zimbabwe  College of Physicians | The major health professional bodies do regulate the practice by health professionals. |
|  | Private sector Industry | Retail Pharmacy Association (RPA) | Association of all community pharmacies. Do determine mark-up levels on medicines sold by community pharmacies to the public. |
|  |  | Pharmaceutical Manufacturers of Zimbabwe | Association of all pharmaceutical manufacturers in Zimbabwe. They are the innovators of health technologies. |
|  |  | Pharmaceutical Wholesalers of Zimbabwe | Association of pharmaceutical wholesalers in Zimbabwe. The have an influence on the supply and price of medicines and diagnostics to private and public health institutions. |
|  |  | Association of Health funders of Zimbabwe (AFHOZ) | Determine tariffs for health services provided by private health institutions and funded by private health insurers. |
|  | Patients | Diabetic Association of Zimbabwe | Lobby for access, price reduction and management of diabetes in Zimbabwe |
|  |  | Zimbabwe National Network of People Living with HIV | Advocate for the provision of treatment and health services for people living with HIV/AIDS |
|  |  | Cancer Association of Zimbabwe | Lobby for access and price reduction of cancer drugs. Also provide funds for cancer drugs. |
| **Producers of Evidence** | Schools of Medicine and Pharmacy | University of Zimbabwe, Harare Institute of Technology, Midlands State University, National University of Science and Technology, Great Zimbabwe University | Universities generate clinical , health economic and health systems evidence relevant for HTA |
|  | Research Institutions | University of Zimbabwe Clinical Trials Centre, Biomedical Research and Training Institute (BRTI), | Research institutes do carry out clinical trials in Zimbabwe providing efficacy evidence for HTA. |
|  | Government based research | Research Council of Zimbabwe, Medical Research Council of Zimbabwe | Provide funding, research agenda and regulation of health research in Zimbabwe |
| **Knowledge Brokers** |  |  |  |
|  | Media | Herald, Zimbabwe Broadcasting Cooperation, Newsday, The Standard Newspaper, Daily News | Print and digital media platforms to have programmes and sections dedicated for health issues and are used to promote health policies. |
| **Funders and International development partners** | UN bodies | WHO-Zimbabwe, UNICEF-Zimbabwe, UNDP-Zimbabwe | International partners provide expertise and capacity building. Also can fund establishment of HTA activities |
|  |  | GAVI, MSF, Global Fund | Provide funding for policy formulation, some health programs. For example vaccines |

UN-United Nations, WHO- World Health Organisation, UNICEF-United Nations International children’s fund, GAVI-Global Alliance for vaccines and Immunisation, MSF-Medecins Sans F

**Figure S1: The thematic coding tree for qualitative data**

**Themes Sub-themes Codes**


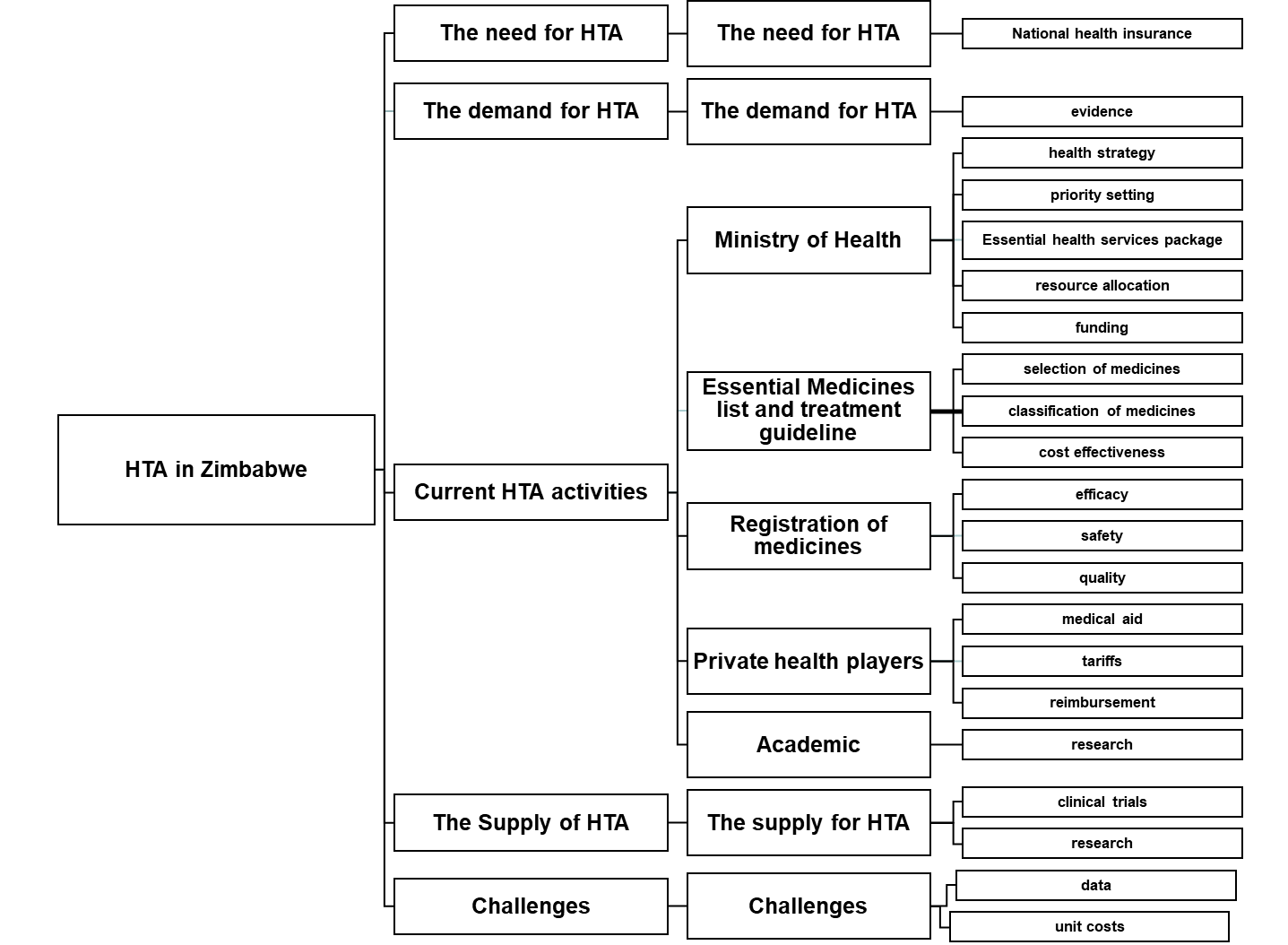


**Table S2: Potential data sources for Health technology assessment in Zimbabwe**

| **HTA data** | **Institution/Data source** | **Data Collection** |
| --- | --- | --- |
| **Epidemiology data** |  |  |
| Demographics and population profile | Zimbabwe National Statistic Agency/Census | Household Survey |
| Disease burden | MoHCC/District Health Information System | Mobile based reporting |
|  | MoHCC/ Zimbabwe Health Profile report | Routine reporting |
| Demographics and health indicators | Zimbabwe National Statistics Agency/Demographic health survey | Household Survey |
| **Clinical effectiveness** |  |  |
| Efficacy | MCAZ/Dossiers | Dossier submissions |
|  | Research Institutes, Universities/Clinical trials | Clinical trials |
| RWE-effectiveness | Not available | Not available |
| Safety | MCAZ/Post marketing surveillance reports | Voluntary reporting |
| **Patient reported outcomes** |  |  |
| QALYs | Universities and Research Institutions | Research |
| DALYs | The Institute for Health Metrics and Evaluation (IHME)/ Global burden of disease |  |
| Patient experiences | Patient groups | Structured collection |
| **Costs** |  |  |
| Health expenditure | MoHCC/ | Routine reporting |
| Health services costs | Health institutions/Service fees | Health services utilisation data |
| Out of pockets costs | AHFOZ | Claims databases of health insurance |
|  |  |  |
| **Health services Utilisation** |  |  |
| Drugs | MoHCC-Department of Pharmacy Services | Routine reporting |
|  | NATPHARM | Reports |
|  | Private pharmacies/ Database | Retail pharmacies databases |
| Diagnostics | MoHCC/Laboratory services | Surveillance |
| Health services coverage | MoHCC/ District Health information system | Mobile based reporting |

MoHCC-Ministry of Health and Child Care, MCAZ-Medicines Control Authority of Zimbabwe, AFHOZ -Association of Healthcare Funders of Zimbabwe, QALYs-Quality adjusted life years, DALYs –Disability adjusted life years.

**The HTA Situational Analysis Questionnaire**

**HTA Situational Analysis Questionnaire**

**Introduction**

*Health Technology Assessment (HTA) is defined as a multidisciplinary field of policy research, which provides evidence on consequences of adoption and use of health technologies. The evidence that is produced by the HTA process is defined as HTA output. Introduction of HTA in countries is driven by three key elements, namely needs for HTA output, demand for HTA output, and supply of HTA output. A conducive situation emerges when the needs, demands and supply are well balanced. This questionnaire was designed to explore the situation concerning HTA in Zimbabwe. This questionnaire* seeks your views on issues relating to HTA specific to Zimbabwe*. If you need clarification on particular feel free to ask the researcher.*

**Name of institution**…………………………………………………………………………………..

**Questions**

| **Current generation and use of evidence**   1. Currently what evidence (clinical effectiveness, health economic, legal, patient reported outcomes) is generated within your organisation and how is being used?   ……………………………………………………………………………………………………………………………………………………………………………………………………………………………………………………………………………………………………………………………………………………………………………………………………………………………………………………………………………………………………………………………………………………………………………………………………………………………………………………………………………………………………………………………………………………………………………………………………………………………………………………………………………………………………………………………………………………………………………………………………………………………………   1. What are the gaps in evidence generation or utilisation?   …………………………………………………………………………………………………………………………………………………………………………………………………………………………………………………………………………………………………………………………………………………………………………………………………………………………………………………………………………………………………………………………………………………………………………………………………………………………………………………………………………………………………………………………………………………………………………………………………………………………………………………………………………….. |
| --- |
| **The Need for HTA**  Due to limited resources as a nation, we can prioritize policy areas for which HTA is needed more than others. This section of the questions requires priority areas for use of HTA in Zimbabwe (e.g. registration of medicines, inclusion into EDLIZ, pricing, reimbursement by medical aids, coverage) |
| 1. HTA is important for the following **attributes**. Tick which are currently **3 most important** for Zimbabwe 2. Ensuring that the money and resources available for health are distributed in the best way to achieve maximum benefits from the money available 3. Transparency in decision making 4. Budget control 5. Equity 6. Improving |
| 1. HTA can be used in many different **health policy areas** to improve the evidence-base used in the decision-making process. Please choose three of the following policy areas, in which the output from a HTA process is urgently needed in Zimbabwe and Why? If you choose more than 1 rank them. 2. Registration of health technologies 3. Coverage or reimbursement of health technologies 4. Production of EDLIZ and clinical pathways 5. Informing design of basic package of health benefits 6. Health service delivery design 7. Provider payment reform or pay for performance schemes |
| 1. Which health technologies need HTA in Zimbabwe. 2. Medicines 3. Vaccines 4. Diagnostics /Medical devices 5. Surgical Procedures 6. Public health programs |
| The demand for HTA output  1.List three organisations in Zimbabwe who are potential consumers of HTA output.  a………………………………………………………………………………………………………………………………………………………b………………………………………………………………………………………………………………………………………………………c………………………………………………………………………………………………………………………………………………………………………………………………………………………………………………………………………………………………………………  2.From the evidence users that you listed in question 1, please rate the level of their interest in different types of HTA outputs. In the grey shaded areas, please use a 0-10 scale where 0 is no demand and 10 is high demand.   \| 0 \| 1 \| 2 \| 3 \| 4 \| 5 \| 6 \| 7 \| 8 \| 9 \| 10 \| \| --- \| --- \| --- \| --- \| --- \| --- \| --- \| --- \| --- \| --- \| --- \|   No demand high demand   \| **Organisation** \|  \| \| \| \| \| \| \| --- \| --- \| --- \| --- \| --- \| --- \| --- \| \|  \|  \| **Safety** \| **Efficacy** \| **Effectiveness** \| **Economics (value for money, costs, budget impact)** \| **Social /ethical concerns** \| \| **a** \| **Level (0-10)** \|  \|  \|  \|  \|  \| \| **b** \| **Level(0-10)** \|  \|  \|  \|  \|  \| \| **c** \| **Level(0-10)** \|  \|  \|  \|  \|  \| |
| **The supply of HTA data**  **Please provide potential sources of HTA input data in Zimbabwe**  Disease epidemiology data  ……………………………………………………………………………………………………………………………………………………………………………………………………………………………………………………………………………………………………………………………………………………………………………………………………………………………………………………………………………………………………………………………………………………………………………………………………………………………………………………………………………………………………………………………………………………………………………………..  Clinical Effectiveness  ……………………………………………………………………………………………………………………………………………………………………………………………………………………………………………………………………………………………………………………………………………………………………………………………………………………………………………………………………………………………………………………………………………………………………………………………………………………………………………………………………………………………………………………………………………………………………………………..  Patient reported outcomes  …………………………………………………………………………………………………………………………………………………………………………………………………………………………………………………………………………………………………………………………………………………………………………………………………………………………………………………………………………………………………………………………………………………………………………………………………………………………….  Costs  …………………………………………………………………………………………………………………………………………………………………………………………………………………………………………………………………………………………………………………………………………………………………………………………………………………………………………………………………………………………………………………………………………………………………………………………………………………………….  Service delivery e.g. health professional salaries  ……………………………………………………………………………………………………………………………………………………………………………………………………………………………………………………………………………………………………………………………………………………………………………………………………………………………………  **Challenges**  In your view what challenges implementation of HTA may face in Zimbabwe  …………………………………………………………………………………………………………………………………………………………………………………………………………………………………………………………………………………………………………………………………………………………………………………………………………………………………………………………………………………………………………………………………………………………………………………………………………………………….  END |
